# Why did EFSA not reduce its ADI for aspartame or recommend its use should no longer be permitted?

**DOI:** 10.1186/s13690-020-00489-w

**Published:** 2020-11-09

**Authors:** Erik Paul Millstone, Elisabeth Dawson

**Affiliations:** SPRU - Science Policy Research Unit, Freeman Centre, Jubilee Building, University of Sussex, Brighton, BN1 9SL England

**Keywords:** Aspartame, Risk assessment, European food safety authority, Rebuttal

## Abstract

On behalf of the European Food Safety Authority (EFSA), Kass and Lodi recently published a letter purporting to ‘refute’ our July 2019 analysis of EFSA’s December 2013 assessment of the risks of aspartame. We had previously claimed inter alia that the EFSA panel had evaluated studies that had indicated that aspartame might be harmful far more sceptically than those that had not indicated harm. We reported that EFSA had deemed every one of 73 studies suggesting harm to have been unreliable. Kass and Lodi provided a tabulation with figures that differed from ours in every detail. This commentary shows that, while Kass and Lodi provided a response to our analysis, they have not come close to refuting it. Our analysis provided detailed characterisations of each of the studies and how the panel interpreted them, but Kass and Lodi provide no corresponding information at all. Kass and Lodi claim that EFSA deemed 21 of 35 studies that had indicated possible harm to have been reliable. But if that is so, we now ask: why did the EFSA panel not recommend that aspartame should be banned, or at least tightly restricted?

**Supplementary Information:**

**Supplementary information** accompanies this paper at 10.1186/s13690-020-00489-w.

## Background

We are grateful to George Kass and Frederica Lodi for their letter, recently published in *Archives of Public Health* [1] in response to the *Archives of Public Health* paper we co-authored entitled ‘EFSA’s toxicological assessment of aspartame: was it even-handedly trying to identify possible unreliable positives and unreliable negatives?’, published in *Archives of Public Health* in July 2019 [2].

### Context

The July 2019 paper in *Archives of Public Health* consisted of three main sections, all of which concerned the putative risks from aspartame. It detailed a complex set of inadequacies that characterised the December 2013 report on aspartame of EFSA’s Panel on Food Additives and Nutrient Sources added to Food (ANS panel) [3].

- The first substantive section reviewed key features of the history of the aspartame saga; it provided detailed documentary evidence of corporate incompetence, dishonesty and a lengthy cover-up, jointly contrived by corporate and official bodies.
- The second section provided a detailed quantitative critique of the panel’s report.
- The third provided a detailed qualitative critique of the evaluative criteria against which the panel evaluated individual studies.

Kass and Lodi’s letter mainly addressed the second of those sections; the first was not addressed at all, while the third was dismissed with an unsupported denial of any lack of even-handedness. We will address their points one at a time.

### The ANS panel’s methodology

One of the contentions of our July 2019 critique of the ANS panel’s report was that the panel’s methodology had differed from that set out in the panel’s report; another was that both the ostensible and the actual methodologies were seriously flawed.

Kass and Lodi’s letter states: “The methodology (defined a priori) for the collection and consideration of the scientific information/data for the risk assessment is detailed in Appendix A of [EFSA’s December 2013 report].” They fail to explain the sense in which the expression ‘a priori’ should be understood. It was certainly not a priori in the conventional sense, i.e. something that is known to be necessarily true without reference to experience [4].

The only intelligible way of interpreting Kass and Lodi’s remark is to treat it as indicating that the methodology was, at an early stage, ***assumed*** to be appropriate, but any such assumption should have been open to revision in the light of new information. Instead, it was treated as an incorrigible given, not open to revision. The December 2013 report of the ANS panel did indeed specify a methodology for the collection of scientific information in an Appendix, but the July 2019 paper provided detailed evidence showing that the actual methodology must have been different, and had not been adjusted in the light of the available evidence.

One response to Kass and Lodi’s assertion that the methodology was set a priori is to ask: prior to what, and when? The methodology had evidently not been decided when EFSA’s **Call for scientific data on Aspartame (E 951)** was issued in June 2011 [5]. Nor had it been set by 14 October 2011, when Hugues Kenigswald, then Head of ANS Unit, wrote to Erik Millstone and requested (letter reference: HK/FL/mp (2011)–out–6,029,860) copies of 27 documents, “… preferably in an electronic form …”.

### Millstone’s October 2011 dossier

On 25 October 2011, Erik Millstone sent the ANS panel’s secretariat a covering letter and a CD-ROM with digital copies of all the 27 requested documents. Copies of those documents are available at http://www.sussex.ac.uk/spru/research/projects/fcs. Those documents provided clear evidence that the reports of at least 15 of G D Searle’s early studies were profoundly unreliable, because the studies had been incompetently conducted and then misleadingly reported. The documents also detailed how those failings had been uncovered, and then covered-up. Nonetheless, the ANS panel’s December 2013 report cited all 15 of those evidently unreliable studies. Thirteen of them were studies of aspartame, while the remaining 2 were studies of its breakdown product known as diketopiperazine (or DKP).

The convention in regulatory toxicology is to refer to studies showing no indication of possible harm as ‘negative’, and studies that do provide indications of harm as ‘positive’. Of the 13 aspartame-related studies, 5 were treated by the panel as if they were reliable negatives, 6 were deemed to be unreliable positives and 2 as unreliable negatives, though not for the reasons evidenced by Millstone’s dossier. The documents provided in the October 2011 dossier were almost entirely excluded from the set of evidence that the panel’s report discussed. But this demonstrates that, when the criteria of inclusion were set, they were set too narrowly. The criteria of inclusion must have been set after that dossier reached EFSA in October 2011; it would have been bizarre if the ANS secretariat had requested information that it already knew would not be deemed worthy of inclusion.

Evidence supporting allegations of scientific incompetence, misleading reporting and a subsequent cover-up certainly deserved inclusion and careful attention. The panel’s criteria for the inclusion of evidence should have been sufficiently broad as to include all of the material that the dossier provided. The documents comprising the dossier are all directly relevant to the substance and reliability of the panel’s assessment of the risks that aspartame might pose. For the evidence that the dossier provided to have been omitted from the panel’s deliberations, or at any rate from their report, was inexcusable.

The ANS panel’s report did cite one of the documents that was included in the dossier, namely the 18 November 1978 report of the Universities Associated for Research and Education in Pathology Incorporated (or UAREP). UAREP pathologists had examined laboratory animal tissue samples on glass slides, which had been provided by G D Searle to the US Food and Drug Administration (FDA), from 12 of the 15 controversial studies. The pathologists compared their interpretations of the slides with those indicated in the reports submitted by Searle to the FDA. However they did not scrutinise the prior actions that had resulted in those tissue samples being placed on those slides. The ANS panel’s December 2013 report (Appendix L pp. 259 et seq) interpreted the UAREP report as having ‘authenticated’ all 12 of the Searle’s early studies, and those of its sub-contractor Hazleton Labs, which it reviewed. However the ANS panel’s report failed to cite the documents provided by the dossier that explained why the UAREP report was flawed, and was itself unreliable [6].

The explanation offered by Kass and Lodi for not including most of the other 26 documents from the dossier was that they were: “… third party anecdotal evidence from e.g. memos, letters or US congressional hearings.” That rationale is profoundly problematic. No fewer than 10 of the items comprising the dosser were official documents of the FDA, and one was the report of a hearing of a US Senate Committee, on 3 November 1987 [7]. To characterise and discount that evidence as ‘anecdotal’ is unacceptable. All the documentary evidence presented to a US Senate hearing, and spoken witness testimony, in the Committee’s report should have been included in the totality of the evidence that the ANS panel reviewed. Instead, to use the expression invoked by Demortain, the ANS panel ‘disenfranchised’ several important sources of information [8]. The panel’s criteria of inclusion of evidence deemed relevant should have included, rather than excluded, all the information and evidence the dossier provided. A priori judgements are often ones that need to be reviewed and reversed in the light of relevant evidence that subsequently becomes available. Priority is not a justification for irresponsible stubbornness.

Dr. Angeliki Lyssimachou, Science Policy Officer at the Pesticides Action Network: Europe, wrote on 4 March 2020 to Stella Kyriakides, EU Commissioner for Health and Food Safety, raising concerns about evidence of scientific fraud at a German laboratory that had been certified as providing ‘Good Laboratory Practice’. [9] Lyssimachou’s allegations about the German laboratory closely resonate with those contained in the October 2011 dossier concerning Searle’s early work. In her response, dated 7 April 2020, Commissioner Kyriakides said, in respect of EFSA’s assessment of active substances and plant protection products, that their assessments: “… must take into account **all** available information.” [10] That surely should be EFSA’s prior criterion; it ought to be a defining characteristic of all EFSA’s risk assessment methodologies, including those of food additives. Practices that deviate from that requirement, such as that exemplified by the ANS panel’s December 2013 report on aspartame, are incompatible with EFSA’s remit.

### The qualitative critique

Our July 2019 paper provided detailed qualitative textual evidence that the ANS panel had judged the reliability of studies suggesting that aspartame might be harmful by far more exacting standards than those it applied to studies that provided no evidence of harm, which were treated far more forgivingly. Kass and Lodi’s letter fails to refer to any of that evidence. Instead, they simply assert that: “… the approach followed by the ANS Panel was a rigorous one, ensuring an independent and in-depth analysis of all existing data...” Their letter failed to provide any evidence to support that assertion, and failed to address the detailed evidence we adduced in support of our contention.

### The quantitative critique

Kass and Lodi’s comments on the quantitative evidence were also entirely unsubstantiated. Our July 2019 paper included a table indicating that the panel had deemed 62 out of 81 ‘negative’ studies as reliable and 19 as unreliable. In contrast, we argued that, of 73 putatively positive studies, the panel had deemed all of them as unreliable. Kass and Lodi’s table suggested that the ANS panel had treated 51 of 78 of negative studies as reliable while 27 were deemed unreliable. They also suggested that 21 of 37 putatively positive studies had been treated by the panel as reliable, while 16 were deemed unreliable. While our July 2019 paper identified each study, and indicated study-specific reasons for our categorisations, Kass and Lodi provided no list, no details and no study-specific reasoning. Furthermore, no explanation was given for the discrepancies between our figures for the total numbers of studies in each of the relevant categories and those provided in Kass and Lodi’s tabulation.

In a letter dated 10 January 2014 Per Bergman, then EFSA’s Head of Regulated Products, responded to correspondence that Millstone had sent a month earlier [11]. Bergman referred to two tables that Millstone had provided, which had indicated estimates of numbers of studies, putatively positive or negative, that the ANS panel had deemed to be either ‘reliable’ or ‘unreliable’ in its ‘Draft Opinion’ on aspartame issued on 8 January 2013. Bergman said: “… in the absence of further information, it is not possible for us to trace back to which individual studies you are referring and how you derived the corresponding numbers for your tables. Therefore, if you are interested to further discuss … as a first step, I would like to kindly ask you to provide the breakdown of the references to the papers and studies that underpin the different figures that you have derived from your tables.”

The paper published last July was, in large part, a response to Bergman’s stipulation that EFSA would not engage further until provided with detailed characterisations of each of the studies and of the ANS panel’s interpretation of them. Our July 2019 paper *did* provide all those details. Unfortunately, Kass and Lodi’s letter provided no corresponding information. It is now, therefore, incumbent on EFSA to provide a detailed breakdown of the studies, and references to the papers, which underpin the figures in Kass and Lodi’s tabulation.

To facilitate EFSA’s work we are providing a tabulated template, as an appendix, see Additional File 1, based on a table provided as Additional File 2 to our July 2019 paper: available as https://static-content.springer.com/esm/art%3A10.1186%2Fs13690-019-0355-z/MediaObjects/13690_2019_355_MOESM2_ESM.pdf . The first two columns in the table we are providing identify the individual studies in the aspartame toxicology section, i.e. Section 3.2, of the ANS Panel’s December 2013 report and the section and page numbers at which the panel’s comments can be found. The third column provides our understanding of the panel’s characterisation of the findings of those studies. Our categorisations are expressed using the same abbreviations as were used in the July 2019 paper, and they are indicated in the Table 1 below. The fourth column is blank and provides the cells into which we invite EFSA to insert its interpretation of those studies; the final column is available for any comments that EFSA may also wish to provide.

**Table 1:** Abbreviations used in tabulation of studies

Abbreviation	Category
rP	Study result(s) deemed reliable by the panel as indicating adverse effects on humans, ie reliable positive
uP	Study result(s) deemed unreliable by the panel as indicating adverse effects on humans, ie unreliable positive
rN	Study result(s) deemed reliable by the panel as indicating no adverse effects on humans, ie reliable negative
uN	Study result(s) deemed unreliable by the panel as indicating no adverse effects on humans ie unreliable negative
Cont	Contradictory, when comparing the wording in Section 3.2 with the text in the appendix
ELlow	Study indicating NOAEL at/or below 4000 mg/kg bw/day
ELhigh	Study indicating adverse effects, but only at doses above 4000 mg/kg bw/day

### Policy implications

Crucially, if, as Kass and Lodi now claim, EFSA’s panel deemed 21 of 37 of ‘positive’ studies (i.e. those indicating harm) as reliable, why did the panel fail to recommend that aspartame should be banned or at least far more tightly restricted? Our detailed analysis located exactly 5 studies [12] that indicated adverse effects, but that were only detected at doses greater than the level of 4000 mg per kilogramme body weight per day (mg/kg bw/day), which the ANS panel designated as the ‘No Observed Adverse Effect Level’ (or NOAEL). NOAELs are important because EFSA sets ‘Acceptable Daily Intake’ (or ADI) levels by dividing NOAELs by a ‘safety factor’, which is conventionally set at 100 [13]. But Kass and Lodi’s figures for the number of studies that the panel deemed to be reliable ‘positive’ studies, i.e. 21 of 37, imply that the panel had identified at least than 16 studies (i.e. 21 minus 5), which it deemed reliable, all of which showed adverse effects at dose levels below 4000 mgs/kg bw [14]. The urgent and inescapable question therefore is: why did the panel not set an ADI below 40 mgs/kg bw, or recommend prohibiting the compound altogether?

We therefore look forward to the publication of a detailed list of all the studies that EFSA now claims were deemed to be reliable positives by the ANS panel in its December 2013 report. For completeness it would also help if EFSA listed all those it deemed to be unreliable positives, as well as reliable and unreliable negatives. We also look forward to learning the answers to:

1. Why did the panel and its secretariat adopt data inclusion criteria that were too narrow to include all the evidence in Millstone’s 2011 dossier?
2. How does EFSA respond to the detailed textual evidence provided in our July 2019 paper demonstrating that the reliability of ‘negative’ studies was judged far more forgivingly than the very exacting standards by reference to which putative ‘positive’ studies were appraised? and
3. What were the panel’s reasons for not interpreting the fact that at least than 16 studies, deemed to be reliable by the panel, showed adverse effects at dose levels below 4000 mgs/kg bw, as providing sufficient grounds for reducing aspartame’s ADI or recommending that its use is no longer permitted?

## Supplementary Information

**Additional file 1.**

## Data Availability

All relevant data and material have already been made available. They can be found at https://static-content.springer.com/esm/art%3A10.1186%2Fs13690-019-0355-z/MediaObjects/13690_2019_355_MOESM1_ESM.pdf and https://static-content.springer.com/esm/art%3A10.1186%2Fs13690-019-0355-z/MediaObjects/13690_2019_355_MOESM2_ESM.pdf

## Abbreviations

ADI: Acceptable Daily Intake
ANS: EFSA’s Panel on Food Additives and Nutrient Sources added to Food
DKP: Diketopiperazine
EFSA: European Food Safety Authority
mg/kg bw/day: Milligrams per kilogramme body weight per day.
NOAEL: No Observed Adverse Effect Level, in mg/kg bw/day
UAREP: Universities Associated for Research and Education in Pathology Incorporated

## References

[1] Kass G, Lodi F (2020). Letter to the editor regarding the article ‘EFSA’s toxicological assessment of aspartame: was it even-handedly trying to identify possible unreliable positives and unreliable negatives?. Arch Public Health.

[2] Millstone E, Dawson E. ‘EFSA’s toxicological assessment of aspartame: was it even-handedly trying to identify possible unreliable positives and unreliable negatives? Arch Public Health. 2019. 10.1186/s13690-019-0355-z. 10.1186/s13690-019-0355-z PMC6628497 31338189

[3] EFSA J. 2013;11(12):3496. available at https://www.efsa.europa.eu/en/efsajournal/pub/3496.

[4] See eg Immanuel Kant, *Critique of Pure Reason*, Riga, 1781, see eg English translation by Norman Kemp Smith, Macmillan Press, London 1968, p 11; P Edwards et al (eds) *The Encyclopedia of Philosophy*, Macmillan, London & New York, 1967, Volume 1, pp. 140–144.

[5] EFSA. Call for Scientific Data on Aspartame (E 951). Parma; 2011. see http://2019Q21.www.efsa.europa.eu/sites/default/files/assets/110601.pdf, Accessed 4 Apr 2019.

[6] eg US FDA Memorandum from Dr Adrian Gross to Mr Carl Sharp at the Food and Drug Administration, 4th November 1976, reproduced in “Nutrasweet” - Health and Safety Concerns, hearing before the committee on Labor and Human Resources of the US Senate, 3rd November 1987, pp. 440-442; Item S. Hrg. 100-567. Washington DC: US Government Printing Office; 1988.

[7] “Nutrasweet” - Health and Safety Concerns, hearing before the committee on Labor and Human Resources of the US Senate, 3rd November 1987, Item S. Hrg. 100-567. Washington DC: US Government Printing Office; 1988.

[8] Demortain D (2017). Expertise, regulatory science and the evaluation of technology and risk. Minerva.

[9] https://www.pan-europe.info/resources/letters/2020/03/open-letter-health-commissioner-kyriakides-fraud-glp-certified. Accessed 14 Apr 2020.

[10] Letter from to Ms A Lysimachou, PAN-Europe, European Commission Reference: Ares(2020)1969076 - 07/04/2020.

[11] Letter from P Bergman, EFSA’s Head of Regulated Products to E Millstone. EFSA; 2014.

[12] Unpublished studies from G D Searle submitted to the US FDA designated as: E33, E34, E70, E14, plus Brunner RL et al, 1979. ‘Aspartame: assessment of developmental psychotoxicity of a new artificial sweetener, Neurobehavioural Toxicology, 1979, 1, 79-86; cf E Millstone & E Dawson, Op Cit Arch Public Health: 2019;77:34. 10.1186/s13690-019-0355-z. Table 3, p 10 and Appendix 2. 551307

[13] Assuming a factor of 10 covers the difference between rodents and humans and another factor of 10 covers the differences amongst humans, see Food Additives and the Consumer, European Commission, 1980, ISBN: 92-825-1232-0.

[14] On the assumption that all of the 21 ‘positive studies’ identified by Kass and Lodi. Arch Public Health. 2020;78:14. 10.1186/s13690-020-0395-4, are included in our set of 73 positive studies, and that their 21 studies include up to five of the studies that we identified as positive, though only at doses greater than 4000 mgs/kg bw/day.

