# Supplementary material for: Why did EFSA not reduce its ADI for aspartame or recommend its use should no longer be permitted?
Source: Arch Public Health. 2020 Nov 9;78:112. doi: 10.1186/s13690-020-00489-w (PMC7654000; doi:10.1186/s13690-020-00489-w)
Supplement: Supplementary file 1 — Additional file 1. [file 13690_2020_489_MOESM1_ESM.docx]

Appendix 1: Comparative tabulation of Millstone & Dawson interpretations of the December 2013 ANS panel's report with EFSA's interpretations

| Citation to source | Location in ANS panel’s report:  Section & page | Millstone & Dawson's understanding of the ANS Panel's categorisations | EFSA's understanding of the ANS Panel's categorisations | Comments |
| --- | --- | --- | --- | --- |
|  | 3.2.1 Acute toxicity of aspartame |  |  |  |
| E46, 1973 | 3.2.1.1 page 56 | rN |  |  |
|  | Appendix F page 202 |  |  |  |
| E84, 1974 | 3.2.1.1 page 57 | rN |  |  |
|  | Appendix F page 202 |  |  |  |
| E85, 1974 | 3.2.1.1 page 57 | rN |  |  |
|  | Appendix F page 202 |  |  |  |
|  | 3.2.2 Short-term and sub-chronic toxicity of aspartame |  |  |  |
| E2, 1972 | 3.2.2.1 page 57 | rN |  |  |
|  | Appendix G page 205 |  |  |  |
| E3, 1972 | 3.2.2.1 page 57 | uP |  |  |
|  | Appendix G page 204 |  |  |  |
| E20, 1969 | 3.2.2.1 page 57 | rN & ELlow |  |  |
|  | Appendix G page 204 |  |  |  |
| E21, 1969 | 3.2.2.1 page 58 | rN |  |  |
|  | Appendix G page 204 |  |  |  |
| Abhilash et al 2011 | 3.2.2.2 page 58 | uP |  |  |
| Abhilash et al 2013 | 3.2.2.2 page 58 | uP |  |  |
|  | 3.2.3. Genotoxicity of aspartame |  |  |  |
| E97 1978 | 3.2.3.1 page 59 | rN |  |  |
|  | Appendix H page 207 |  |  |  |
| E101 1978 | 3.2.3.1 page 59 | rN |  |  |
|  | Appendix H page 207 |  |  |  |
| E81 1974 | 3.2.3.1 page 59 | uN & Cont |  |  |
|  | Appendix H page 210 | Cont |  |  |
| E40 1973 | 3.2.3.1 page 59 | rN |  |  |
|  | Appendix H page 209 |  |  |  |
| E41 1973 | 3.2.3.1 page 59 | rN |  |  |
|  | Appendix H page 209 |  |  |  |
| E43 1972 | 3.2.3.1 page 59 | rN |  |  |
|  | Appendix H page 209 |  |  |  |
| E12 1970 | 3.2.3.1 page 60 | uN |  |  |
|  | Appendix H page 210 |  |  |  |
| E44 1972 | 3.2.3.1 page 60 | uN & Cont |  |  |
|  | Appendix H page 210 | Cont |  |  |
| NTP 2005 | 3.2.3.2 page 60 | rN |  |  |
|  | Appendix H page 207 |  |  |  |
| Rencuzogullari et al 2004 | 3.2.3.2 page 60 | uN |  |  |
|  | Appendix H page 208 |  |  |  |
| Bandyopadhyay et al 2008 | 3.2.3.2 page 60 | uN |  |  |
| Jeffrey and Williams 2000 | 3.2.3.2 page 60 | rN |  |  |
|  | Appendix H page 208 |  |  |  |
| Rencuzogullari et al 2004 CA | 3.2.3.2 page 60 | uP |  |  |
| Rencuzogullari et al 2004 MN |  | uP |  |  |
| Rencuzogullari et al 2004 SCE |  | uN |  |  |
|  | Appendix H page 208 |  |  |  |
| Durnev et al 1995 | 3.2.3.2 page 61 | uN |  |  |
|  | Appendix H page 211 |  |  |  |
| Mukhopadhyay et al 2000 | 3.2.3.2 page 61 | uN |  |  |
|  | Appendix H page 211 |  |  |  |
| NTP 2005 micronucleus | 3.2.3.2 page 61 | rN |  |  |
|  | Appendix H page 211 |  |  |  |
| NTP 2005 (p53-haploinsufficient) | 3.2.3.2 page 61 | uP in haploinsufficient, but rN in other mice strains |  |  |
| NTP 2005 (TG.AC hemizygous) | 3.2.3.2 page 61 | rN |  |  |
| NTP 2005 (CDkn2A deficient) | 3.2.3.2 page 61 | rN |  |  |
|  | Appendix H page 212 |  |  |  |
|  | Appendix H page 212-213 |  |  |  |
| Sasaki et al 2002 | 3.2.3.2 page 62 | rN |  |  |
|  | Appendix H page 213 |  |  |  |
| Bandyopadhyay et al 2008 | 3.2.3.2 page 62 | uP |  |  |
|  | Appendix H page 213 |  |  |  |
| Kamath et al 2010 | 3.2.3.2 page 62 | uP |  |  |
|  | Appendix H page 213/214 |  |  |  |
| AlSuhaibani 2010 CA | 3.2.3.2 page 62 | uP |  |  |
| AlSuhaibani 2010 SCE |  | uN |  |  |
|  | Appendix H page 214 |  |  |  |
| Karikas et al 1998 | 3.2.3.2 page 63 | uP |  |  |
| Meier et al 1990 | 3.2.3.2 page 63 | uP |  |  |
| Shephard et al 1993 | 3.2.3.2 page 63 | uP |  |  |
|  | 3.2.4 Chronic toxicity and carcinogenicity of aspartame |  |  |  |
| E75 1974 | 3.2.4.1 page 64 | uP |  |  |
| E33-34 1973 | 3.2.4.1 page 64 | ELhigh |  |  |
| E70 1974 | 3.2.4.1 page 65 | uP and ELhigh |  |  |
| E87 1973 (brains from E33-34 and E70) | 3.2.4.1 page 66 | rN |  |  |
| Ishii et al 1981 | 3.2.4.1 page 68 | uP and ELlow |  |  |
| E27 1972 | 3.2.4.1 page 68 | uN |  |  |
| E35-36 1972 | 3.2.4.1 page 68 | uN |  |  |
| E28 1972 | 3.2.4.1 page 69 | uN |  |  |
| E86 1973 | 3.2.4.1 page 69 | rN |  |  |
| E32 1972 = Waisman study | 3.2.4.1 page 69 | uP |  |  |
| Soffritti et al 2006 | 3.2.4.2 page 70 | uP |  |  |
| Soffritti et al 2007 | 3.2.4.2 page 70 | uP |  |  |
| Soffritti et al 2010 | 3.2.4.2 page 71 | uP |  |  |
| NTP 2005 | 3.2.4.2 page 71 | rN - counted above |  |  |
|  | 3.2.5 Reproductive and developmental toxicity of aspartame |  |  |  |
| E11 1971 | 3.2.5.1.1 page 72 | uP and ELlow |  |  |
|  | Appendix I page 227 |  |  |  |
| E9 1972 | 3.2.5.1.1 page 72 | uP and ELlow |  |  |
|  | Appendix I page 227 |  |  |  |
| E10 1972 | 3.2.5.1.1 page 72 | uP |  |  |
|  | Appendix I page 227, column 6 |  |  |  |
| E39 1973 | 3.2.5.1.1 page 73 | uP and ELlow |  |  |
|  | Appendix I page 227-8 |  |  |  |
| E88 1975 | 3.2.5.1.1 page 73 | uP |  |  |
| E89 1975 | 3.2.5.1.2 page 73 | rN |  |  |
|  | Appendix I page 227 |  |  |  |
| E47 1973 | 3.2.5.1.2 page 74 | uP, Cont and ELlow |  |  |
|  | Appendix I page 228 | Cont |  |  |
| E48 1973 | 3.2.5.1.2 page 74 | uP and Cont |  |  |
|  | Appendix I page 228 | Cont |  |  |
| E49 1973 | 3.2.5.1.2 page 74 | uP |  |  |
| E5 1970 | 3.2.5.1.2 page 74 | uP and Cont |  |  |
|  | Appendix I page 227 | Cont |  |  |
| E53 1973 | 3.2.5.1.2 page 75 | uP, Cont, ELlow |  |  |
|  | Appendix I page 227 | Cont |  |  |
| E54 1974 | 3.2.5.1.2 page 75 | uP and ELlow |  |  |
|  | Appendix I page 227 |  |  |  |
| E55 1973 | 3.2.5.1.2 page 75 | uP and ELlow |  |  |
|  | Appendix I page 229 |  |  |  |
| E62 1973 | 3.2.5.1.2 page 75 | uP |  |  |
|  | Appendix I page 229 |  |  |  |
| E63, 1973 | 3.2.5.1.2 page 75 | uP, Cont and ELlow |  |  |
|  | Appendix I page 229 | Cont |  |  |
| E51 1973 | 3.2.5.1.2 page 76 | uP, Cont and ELlow |  |  |
|  | Appendix I page 230 | Cont |  |  |
| E52 1973 | 3.2.5.1.2 page 76 | uP, Cont and ELlow |  |  |
|  | Appendix I page 230 | Cont |  |  |
| E79 1974 | 3.2.5.1.2 page 76 | uP, Cont and ELlow |  |  |
|  | Appendix I page 230 | Cont |  |  |
| E90 1975 | 3.2.5.1.2 page 76 | uP and ELlow |  |  |
|  | Appendix I page 231 |  |  |  |
| Brunner et al 1979 | 3.2.5.2.1 page 78 | ELhigh |  |  |
| Lennon et al 1980 | 3.2.5.2.1 page 78 | uN |  |  |
| Lennon et al 1980 | 3.2.5.2.1 page 78 | uN |  |  |
| Mahalik and Gautieri 1984 | 3.2.5.2.2 page 79 | uP |  |  |
| McAnulty et al 1989 | 3.2.5.2.2 page 79 | uP and ELlow |  |  |
| Holder 1989 | 3.2.5.2.2 page 79/81 | rN |  |  |
| NTP-CERHR Report 2003 | 3.2.5.2.2 page 79 | rN & ELlow |  |  |
| Collison et al 2012a | 3.2.5.2.2 page 80 | uP |  |  |
| Collison et al 2012b | 3.2.5.2.2 page 80 | uP |  |  |
| Lennon et al 1980 | 3.2.5.2.2 page 81 | ELhigh |  |  |
| Ranney et al 1975 | 3.2.5.2.2 pages 81-82 | uP |  |  |
|  | 3.2.6 Other studies on aspartame |  |  |  |
| E94 year not reported | 3.2.6.1 page 82 | uP |  |  |
| Reynolds et al 1976 | 3.2.6.1 page 82 | uP |  |  |
| E14 1972 | 3.2.6.1 page 82 | uP, ELhigh |  |  |
| Beck et al 2002 | 3.2.6.1 page 83 | uP |  |  |
| Christian et al 2004 | 3.2.6.1 page 83 | uP |  |  |
| Puica et al 2008 | 3.2.6.1 page 83 | uP |  |  |
| Puica et al 2009 | 3.2.6.1 page 83 | uP |  |  |
| E104 1979 | 3.2.6.1 page 83 | rN |  |  |
| Reynolds et al 1980 | 3.2.6.1 page 83 | rN |  |  |
| E105 year not reported | 3.2.6.1 page 83 | rN |  |  |
| Magnuson 2007 | 3.2.6.1 page 84 | rN |  |  |
| EFSA 2010 Review | 3.2.6.1 page 84 | rN |  |  |
| SCF 2002 Review | 3.2.6.1 page 84 | rN |  |  |
| E15 1972 | 3.2.6.2 page 84 | uN |  |  |
| Tutelyan et al 1990 | 3.2.6.2 page 84 | uP |  |  |
| Vences-Mejia et al 2006 | 3.2.6.2 page 84 | uP |  |  |
| Alleva et al 2011 | 3.2.6.3 page 84 | uP |  |  |
| Haque and Mozaffar 1993 | 3.2.6.3 page 84 | uN |  |  |
| Simintzi et al 2007a | 3.2.6.3 page 85 | uP |  |  |
| Simintzi et al 2007b | 3.2.6.3 page 85 | uP |  |  |
| Kim et al 2011 | 3.2.6.3 page 86 | uP |  |  |
| E1 1972 | 3.2.6.3 page 86 | rN |  |  |
| E19 year not provided | 3.2.6.3 page 86 | rN |  |  |

|  | 3.2.7 Human studies of aspartame |  |  |  |
| --- | --- | --- | --- | --- |
| SCF 2002, Review | 3.2.7.1 p 86 | rN |  |  |
| Halldorsson et al 2010 | 3.2.7.1 page 86 | uP |  |  |
| Englund-Ögge et al 2012 | 3.2.7.1 page 87 | uP |  |  |
| La Vecchia 2013 - meta-analysis of Halldorsson et al (2010) and Englund-Ögge et al (2012) | 3.2.7.1 page 88 | uP |  |  |
| Maslova et al 2013 | 3.2.7.1.2 page 88 | uP |  |  |
| Hardell et al 2001 | 3.2.7.1.3 page 89 | uP |  |  |
| Bunin et al 2005 | 3.2.7.1.3 page 89 | uP |  |  |
| Gallus et al 2006 | 3.2.7.1.3 page 90 | uN |  |  |
| Bosetti et al 2009 | 3.2.7.1.3 page 90 | uN |  |  |
| Andreatta et al 2008 | 3.2.7.1.3 page 91 | uP |  |  |
| Lim et al 2006 | 3.2.7.1.3 page 91 | rN |  |  |
| Cabaniols et al 2011 | 3.2.7.1.3 page 91 | uN |  |  |
| Schernhammer et al 2012 | 3.2.7.1.3 page 92 | uP |  |  |
| E66 1973 | 3.2.7.2 page 92 | rN |  |  |
|  | Appendix J page 232 |  |  |  |
| E110 1979 | 3.2.7.2 page 92 | rN |  |  |
| E23 1972 | 3.2.7.3 page 92 | rN |  |  |
| E24 1972 | 3.2.7.3 page 93 | rN |  |  |
| E60 1973 | 3.2.7.3 page 93 | rN |  |  |
| E61 1972 | 3.2.7.3 page 93 | rN |  |  |
| E95 1977 | 3.2.7.3 page 94 | rN |  |  |
|  | Appendix J page 233 |  |  |  |
| Leon et al 1989 | 3.2.7.3 page 94 | rN |  |  |
| Porikos and Van Italie 1983 | 3.2.7.3 page 94 | rN |  |  |
| E25 1972 | 3.2.7.3 page 94 | rN |  |  |
| E67 1973 | 3.2.7.3 page 95 | rN |  |  |
|  | Appendix J page 238 |  |  |  |
| E109 1978 | 3.2.7.3 page 95 | rN |  |  |
| E26 1972 | 3.2.7.3 page 95 | rN |  |  |
|  | Appendix J page 242 |  |  |  |
| Krusei et al 1987 | 3.2.7.4 page 96 | rN |  |  |
| Wolrach et al 1984 | 3.2.7.4 page 96 | rN |  |  |
| Shaywitz et al 1994a | 3.2.7.4 page 96 | rN |  |  |
| Roshon and Hagen 1989 | 3.2.7.4 page 96 | rN |  |  |
| Saravis et al 1990 | 3.2.7.4 page 97 | rN |  |  |
| Lapierre et al 1990 | 3.2.7.4 page 97 | rN |  |  |
| Ryan-Harshman et al 1987 | 3.2.7.4 page 97 | rN |  |  |
| Pivonka & Grunewald 1990 | 3.2.7.4 page 97 | rN |  |  |
| Stokes et al 1991 | 3.2.7.4 page 97 | rN |  |  |
| Stokes et al 1994 | 3.2.7.4 page 97 | rN |  |  |
| Walton et al 1993 | 3.2.7.4 page 97 | uP |  |  |
| Spiers et al 1998 | 3.2.7.4 page 98 | rN |  |  |
| Camfield et al 1992 | 3.2.7.5 page 98 | uP |  |  |
| Shaywitz et al 1994b | 3.2.7.5 page 99 | rN |  |  |
| Rowan et al 1995 | 3.2.7.5 page 99 | rN |  |  |
| Schiffman et al 1987 | 3.2.7.6 page 99 | rN |  |  |
| Koehler and Glaros 1988 | 3.2.7.6 page 99, 101 | uP |  |  |
| Lipton et al 1989 | 3.2.7.6 page 99, 101 | uP |  |  |
| Van den Eeden et al 1994 | 3.2.7.6 page 100, 101 | uP |  |  |
| Szucs et al 1986 | 3.2.7.8 page 100 | rN |  |  |
| Kulczycki 1986 | 3.2.7.8 page 100 | uP |  |  |
| Garriga et al 1991 | 3.2.7.8 page 100 | rN |  |  |
| Geha et al 1993 | 3.2.7.8 page 100 | rN |  |  |
| Butchko et al 2002 Review | 3.2.7.8 page 101 | uP |  |  |
| Novick 1985 | 3.2.7.8 page 101 | uP |  |  |
| McCauliffe and Poitras 1991 | 3.2.7.8 page 101 | uP |  |  |
| Veien and Lomholt 2012 | 3.2.7.8 page 101 | uP |  |  |
| Robert 2001, reviewed by EFSA 2010 | 3.2.7.9 page 101 | uP |  |  |
